# Diversity and Breadth of Host Specificity among Arthropod Pathogens in the *Entomophthoromycotina*

**DOI:** 10.3390/microorganisms11071658

**Published:** 2023-06-26

**Authors:** Natalie E. Sacco, Ann E. Hajek

**Affiliations:** Department of Entomology, Cornell University, Ithaca, NY 14853, USA; nes82@cornell.edu

**Keywords:** entomopathogenic fungi, host range, specialist, generalist, niche breadth evolution, terrestrial fungi, *Zoopagomycota*, *Zygomycota*, insect pathogen, *Entomophthorales*

## Abstract

A meta-analysis based on the published literature was conducted to evaluate the breadth of host ranges of arthropod pathogens in the fungal subphylum *Entomophthoromycotina*. The majority of pathogens in this subphylum infect insects, although arachnids (especially mites), collembola, and myriapods are also used as hosts. Most species (76%) have specialized host ranges and only infect arthropods in one host family. The breadth of host ranges in the *Entomophthoromycotina* is generally greater for species in more basal groups (*Conidiobolaceae* and *Neoconidiobolaceae*), where most species are soil-borne saprobes and few are pathogens. The *Batkoaceae* is a transitionary family in which all species are pathogens and both generalists and specialists occur. Among pathogen-infecting insects, Hemiptera and Diptera are the most commonly infected insect orders. Within the Hemiptera, hosts in the suborder Sternorrhycha were infected by more fungal species than the Auchenorrhyncha and Heteroptera.

## 1. Introduction

Fungal pathogens infecting arthropod hosts vary from specialists to generalists [1], with specialists being finely tuned to their hosts. It is hypothesized that having little to no host diversity leads to a pathogen’s greater success in infection, growth, reproduction, and dispersal, allowing specialists to possess an increased fitness when compared to generalists [2]. However, generalists may have better chances of transmission due to an increased availability of potential hosts. Thus, in cases where there is no cost to generalism (i.e., equal fitness in using few vs. many hosts), being a generalist can result in an overall greater fitness as well as a greater genetic diversity for the pathogen [2]. As a real-world example exploring the costs vs. benefits of generalism, the entomophthoralean fungus *Batkoa major* has a broad host range [3], including the invasive planthopper, *Lycorma delicatula*, which can be present in large numbers, thus providing many hosts to infect [4]. Yet, the fitness of *B. major* is not optimal per individual host when infecting *L. delicatula*. Therefore, although *B. major*’s broad host range provides more opportunities for infection, there is a trade-off to fungal fitness because *L. delicatula* is an abundant yet suboptimal host.

Species in the fungal subphylum *Entomophthoromycotina* range from saprobes to pathogens. Among the pathogens, while a few species infect desmid algae (infected by *Ancylistes*), fern gametophytes (*Completoria*), and nematodes and tardigrades (*Neoconidiobolus* and *Meristacrum*) [5,6], most species infect arthropods. A majority of arthropod hosts belong to the class Insecta [7], with infections also found in the class Arachnida and a few known from the class Entognatha, and the subphylum Myriapoda. Arthropod-pathogenic species in the fungal order *Entomophthorales*, the largest order within the *Entomophthoromycotina*, have often been characterized as having narrow host ranges [8,9,10,11]. However, broader host ranges for some species have also been reported, e.g., *B. major* [3], *Conidiobolus coronatus* [12], and *Zoophthora radicans* [13]. Interest in these pathogens has often focused on their potential for pest control. This type of application would require knowledge of the breadth of hosts infected by these fungi [14,15] as to avoid any unintended impacts on native or beneficial biodiversity. 

The majority of arthropod pathogens in the *Entomophthoromycotina* are obligate, causing acute infections that kill infected hosts relatively quickly, resulting in the discharge of ballistic conidia. Many species in the *Entomophthorales* are well known for their ability to create dramatic epizootics [8,9,10]. Some of these species also change the behavior of infected insects to increase spore dispersal [16]. Behaviors are changed either directly before death [17,18,19] or while an infected arthropod is alive [20,21,22]. In depth studies focused on the genetics and population structure among *Entomophthora* species [23,24,25,26,27,28], the *Entomophaga aulicae* species complex [29], and the *Entomophaga grylli* species complex [30,31], have demonstrated the genetic variability within species, the occurrence of cryptic species, and the variability in host specificity among closely related species. 

Entomophthoralean fungi can be difficult to find and collect, as their occurrences are often seasonal, localized, and ephemeral [8]. Many species have only been identified based on morphological features. Species that have been cultured are usually fastidious, but many have never been cultured. Partial gene and intron sequences are available for relatively few species [32] and, at present, the genomes of very few species have been sequenced. Those that have been sequenced have among the largest genomes of any fungi [33]. Host range was an important feature in defining species within this group prior to the advent of molecular information and remains important today, as available sequences are limited. Once detailed information becomes available for more species, those which are considered individual species today in some cases might be divided, while in other cases, numerous extant species might be merged. Regardless, new species are described every year (e.g., Keller et al. [34] and Eilenberg et al. [35]) which is consistent with the suggestion that many more fungal species remain to be discovered and described [36]. 

We conducted a meta-analysis to investigate the breadth of host range of arthropod pathogens in the *Entomophthoromycotina*. A recent paper surveying the patterns of host specificity in the *Entomophthoromycotina* included 84 species for such an analysis, which is only a part of the recognized species [7]. This paper used host records from the literature and from culture collection databases. Our study included all valid species of arthropod pathogens for which family-level data on hosts were available (=246 species). We based our meta-analysis on published host ranges, evaluating the breadth and diversity of host ranges for all arthropod-pathogenic species in the *Entomophthoromycotina*.

## 2. Materials and Methods

A primary source of host range information for this meta-analysis was the study conducted by Balazy [9], whose work was supplemented by Zha et al. [37], and that of Kirk [38]. All references used as primary sources for data on hosts are listed in Supplementary Materials, SM1. Arthropod-pathogenic species in the subphylum *Entomophthoromycotina* (phylum *Zoopagomycotina* [39]) were included (Table S1), except as follows. For species within the *Entomophthoromycotina*, we used valid combinations of generic and specific names based on Mycobank [40] and Index Fungorum [38]. We excluded fungal species for which the taxonomy seemed questionable (*n* = 11) (Table S2). 

For taxonomy of hosts, we followed naming conventions from GBIF [41], Giribet and Edgecombe [42], Turnbull and Stebaeva [43], and Krantz and Walter [44]. Clear identification of hosts to family level was required for inclusion; for example, hosts of some fungal species were only identified in the literature to class or order (*n* = 13) and these were excluded from analyses (Table S2). 

The primary goal of this study was to investigate the breadth of relationships of entomophthoromycotinan pathogens with hosts. We asked whether a fungal species was pathogenic to: (1) host species within one host family, (2) host species within more than 1 host family within the same host order, (3) host species within 2 host orders, or (4) host species within >2 host orders. Host lists within each of these categories are not exhaustive; our aim was to identify the taxonomic diversity of hosts based on these categories. Therefore, we did not look for every reference to host species within the same host family. Additionally, after we found that a fungal species infected hosts in two families within one order, we principally searched for records of infections by that species in other host orders. Therefore, the data we used (see Table S1) cannot be assumed to include a complete list of host families or orders infected by any of these fungal species. As experimental manipulations can expand host ranges compared with specificity occurring in nature [45], only naturally occurring infections were included. Our criteria for inclusion resulted in a total of 246 arthropod-pathogenic species. 

**Figure 1:**
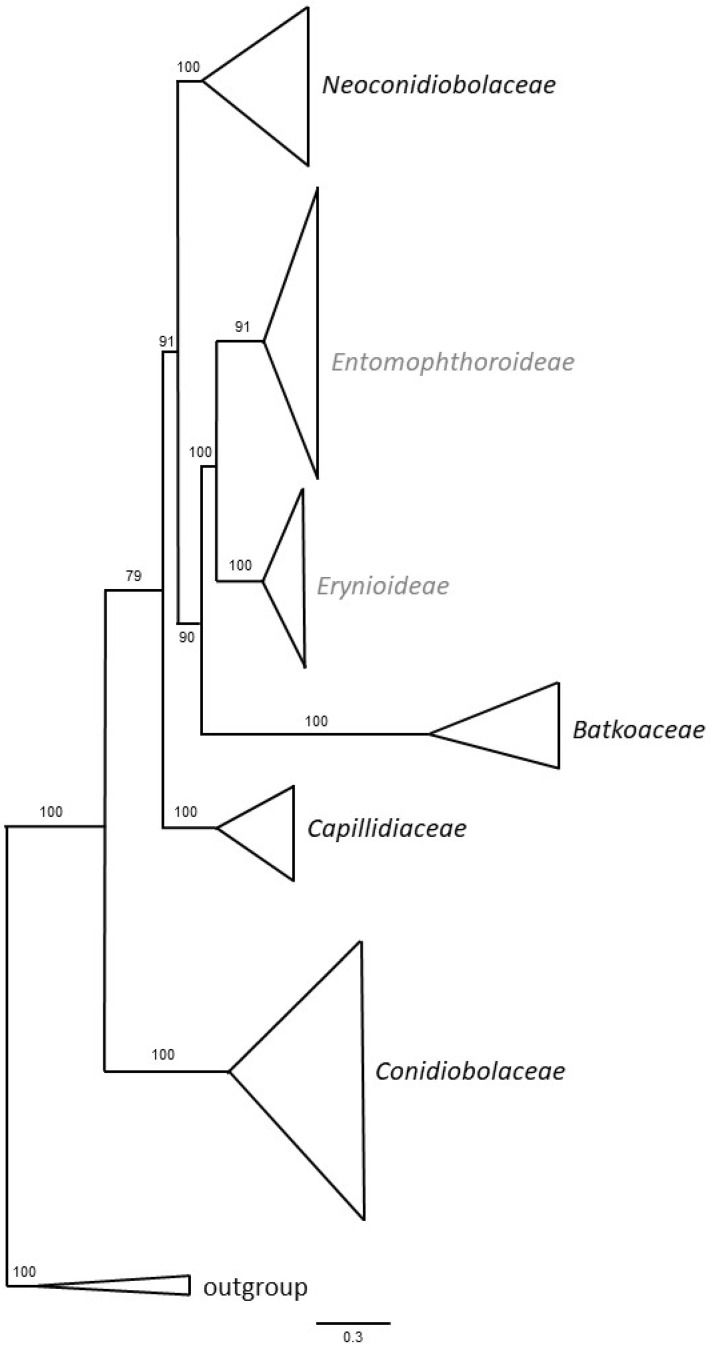
Families and subfamilies with arthropod pathogens in the order *Entomophthorales*, class *Entomophthoromycetes*. The order *Neozygitales* is not included because it is in a separate class. Subfamilies within *Entomophthoraceae* are in grey. Maximum likelihood tree from Gryganskyi et al. [47], modified. Bootstrap values are shown at the basal nodes.

For analyses, pathogens were grouped by family, except the largest family, the *Entomophthoraceae*, which was evaluated using the two subfamilies: *Erynioideae* and *Entomophthoroideae* (Table 1; Figure 1). The informal genus *Tarichium* is a special case because these species are only known from resting spores (zygospores or azygospores) and many were named before molecular methods were available. Without DNA sequences, identifications to the correct fungal genus and family are not possible for species in *Tarichium*. Species formerly in *Tarichium* for which sequences are available have already been moved to the appropriate genera [46]. Therefore, although species in the genus *Tarichium* have been included in parts of our analyses, we cannot say to what extent species within this group are related. This quandary can be overcome in the future as sequences become available.

## 3. Results

**Figure 2:**
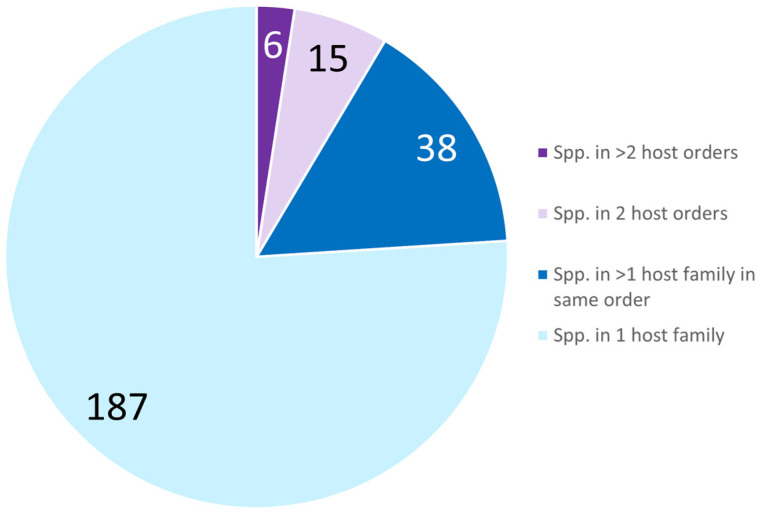
Numbers of arthropod-pathogenic species in the *Entomophthoromycotina* with different breadths of host range.

The majority of entomophthoromycotinan arthropod pathogens only infect hosts in one taxonomic family (76.0%) (Figure 2; Table S1). A much smaller percent (15.5%) of species have been reported from >1 host family within the same host order. Fewer fungal species (8.5%) were reported infecting hosts from >1 order. This last group includes sixteen species, of which all but six were known from hosts in only two orders. The six outliers with the broadest host ranges include *B. major, Batkoa apiculata* in the *Batkoaceae*, *C. coronatus* in the *Conidiobolaceae*, *Neoconidiobolus osmodes* and *Neoconidiobolus thromboides* in the *Neoconidiobolaceae*, and *Z. radicans* in the *Erynioideae*, all reported infecting > 2 insect orders. Among these six species with the broadest host ranges, only *C. coronatus* infects arthropods outside of the class Insecta as well as infecting insects. Most species infecting multiple orders were in the more basal families [47]: *Conidiobolaceae*, *Neoconidiobolaceae*, and *Batkoaceae* (Table 1). However, a few species with hosts in two insect orders were found across *Entomophthoraceae*, *Neozygitaceae*, and in *Tarichium*.

The majority of the 17 genera in the *Entomophthoromycotina* infect diverse hosts. For example, the most speciose genus, *Zoophthora* (38 species; Table S1), includes hosts in eight insect orders. As seeming exceptions, the 12 species in the genus *Massospora* are only known from host species in the family Cicadidae (order Hemiptera) [48]. The genus *Strongwellsea* specializes on dipterans in the subsection Schizophora within the Brachycera, with eight of the nine species infecting hosts in the calyptrate subfamily Muscoidea, and one species known only from an acalyptrate host [49,50]. 

**Figure 3:**
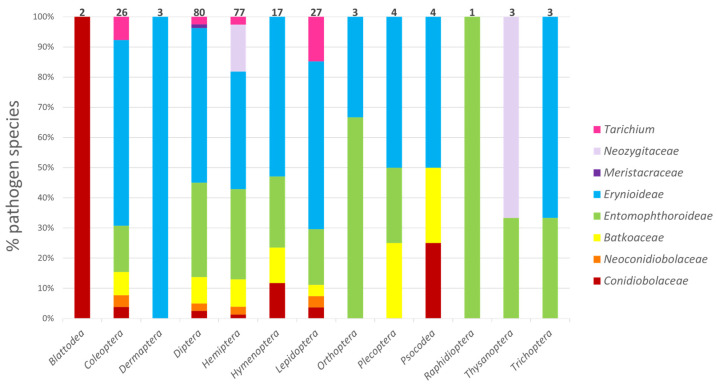
Relative abundance of entomophthoromycotinan species infecting different insect orders by fungal families and subfamilies. Total numbers of arthropod-pathogenic species within the *Entomophthoromycotina* infecting differing insect orders are provided above bars.

The most commonly infected insect orders were the Diptera (80 fungal species) and Hemiptera (77) (Figure 3). The next most common host orders infected were Lepidoptera (27 fungal species), Coleoptera (26), and Hymenoptera (17). Entomophthoromycotinan species have been reported infecting eight additional insect orders, although only a few fungal species were recorded infecting each of these orders. Among the five most commonly infected insect orders, there does not appear to be any strong specialization by families within the *Entomophthoromycotina*; each of these insect orders is infected by fungal species from diverse families. For Blattodea, Dermaptera, and Raphidioptera, the lack of diversity in pathogens could be due in part to the fact that these are less speciose orders.

**Figure 4:**
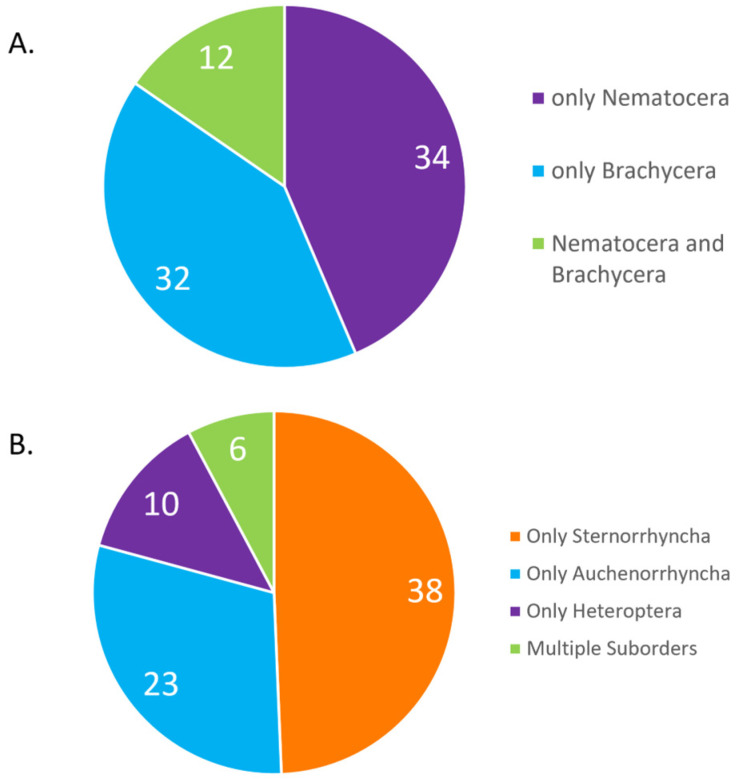
(**A**) Numbers of entomophthoromycotinan species known to infect specific suborders within Diptera. (**B**) Numbers of entomophthoromycotinan species known to infect specific suborders within Diptera.

We investigated pathogen species’ prevalence in the suborders within Diptera and Hemiptera. For Diptera, there was no difference in the number of fungal species known to infect Nematocera versus Brachycera (χ^2^ = 0.0191; P = 0.889991) (Figure 4A). Within Hemiptera, there were fewer fungal species infecting Heteroptera compared with either Auchenorrhyncha (χ^2^ = 10.5564, P = 0.001158) or Sternorrhyncha (χ^2^ = 28.5257, P < 0.00001) (Figure 4B). More fungal species infected Sternorrhyncha than Auchenorrhyncha (χ^2^ = 5.0389; P = 0.024784). By investigating only species in the *Erynioideae*, the numbers of species infecting Hemiptera (30) versus Diptera (41) did not differ significantly (χ^2^ = 3.4085, P = 0.64863).

**Figure 5:**
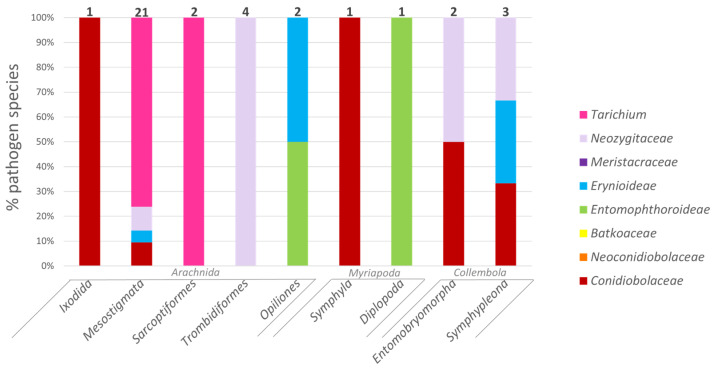
Relative abundance of entomophthoromycotinan species infecting different non-insect arthropods by fungal families and subfamilies. Total numbers of non-insect arthropod-pathogenic species within the *Entomophthoromycotina* infecting differing insect orders are provided above bars.

Although the majority of entomophthoromycotinan species infect only insects, 30 species (including those in *Tarichium*) infect non-insect arthropods (Figure 5). The pathogen species with the most non-insect hosts was *C. coronatus*, which has been reported infecting hosts in six insect orders as well as a tick (class Arachnida), a myriapod (class Symphyla), and species in two orders of collembolans (class Entognatha) (Table S1). Aside from *C. coronatus*, fungi infecting arthropods outside of the class Insecta often specialize in a certain group; for example, *Arthrophaga myriapodina* is only known to infect millipedes (Diplopoda) and *Entomophaga batkoi* only infects opilionids (Arachnida). Among the twenty-one species in the *Neozygitaceae*, one species infects two orders of collembolans and six species infect mites, while the remainder infect Hemiptera and Thysanoptera. Among the 27 species of the form-genus *Tarichium*, 16 species infect mites (see Discussion). 

## 4. Discussion

The majority of arthropod pathogens in the *Entomophthoromycotina* have very narrow host ranges (Figure 2) and this is most pronounced in the most speciose family, the *Entomophthoraceae* (Table 1). Arthropod pathogens within the families *Conidiobolaceae*, *Neoconidiobolaceae*, and *Batkoaceae* contain higher percentages of species with broader host ranges. These latter families are more basally divergent, based on recent phylogenetic analyses [47] (Figure 1). This result confirms that with increasing species divergence within the *Entomophthoromycotina*, there was a trend toward specialism versus generalism. This trend would agree with the long-standing evolutionary theory that parasites evolve toward host specialization [51]. An outlier would be *Z. radicans*, a species in the *Erynioideae* subfamily of the *Entomophthoraceae*, that has a worldwide distribution and a broad host range (five insect orders). While it has been suggested that *Z. radicans* constitutes a species complex [9], a recent study including isolates of *Z. radicans* from diverse families in the Hymenoptera, Hemiptera, Diptera, and Lepidoptera reported that this species is monotypic and not a species complex [13]. This result is consistent with results from a species in the *Batkoaceae*, *Batkoa major*, which has also been shown to be monotypic although a generalist [3]. However, since all other species in the genus *Zoophthora* are more specialized, this appears to be an evolutionary change from specialism to generalism. Studies have shown that evolutionary changes from specialism to generalism are not uncommon (e.g., [52,53]), and some studies have also suggested that host specialization can be a dynamic trait without constant directionality [54]. 

The high percentage of entomophthoromycotinan species with restricted host ranges would suggest that, in this group, this strategy has proven to be superior to a more generalized host range. Theory suggests that there are trade-offs for pathogens adopting specialism versus generalism: traits that increase transmission and fitness when infecting only one host species or multiple closely related hosts (as could be more typical of specialists) may come at a cost, leading to decreased transmission and fitness in other hosts if infection is even possible [2]. Woolhouse et al. [2] suggest that most ‘specialist’ pathogens are not exclusive to one host species, allowing a limited degree of flexibility to facilitate persistence. However, with this limited flexibility, there is the concern that pathogen specialization could lead to extinction (a ‘dead end’) if opportunities to persist (i.e., infect new hosts) do not occur, as when host species are rare. Unlike specialists, generalists would have greater opportunities to locate potential hosts and therefore have increased abilities to persist in the community due to their broader host ranges. Nevertheless, generalists must retain the ability to successfully infect and reproduce in multiple hosts, which could result in a decreased fitness in different host species (e.g., Hajek et al. [4]) compared with specialists. Perhaps the production of resting spores by most species in the *Entomophthoromycotina* [55], or the use of other specialized means of persistence [56,57,58], provide many species in the *Entomophthoromycotina* with the ability to succeed as specialists and persist without the necessity of infecting diverse hosts. In addition, while this study only evaluated specificity to the family level and not below, the use of closely related species within the same family could also facilitate the persistence of specialists.

A recent paper by Möckel et al. [7], based on 84 species in the *Entomophthoromycotina*, stated that the most common hosts of *Entomophthoromycotina* are hemipterans. In contrast, our study, based on the published accounts of 246 arthropod-pathogenic species in the *Entomophthoromycotina*, found that hemipterans and dipterans were equally abundant as the most common hosts (Figure 3), in agreement with Keller and Wegensteiner [10]. Möckel et al. [7] also stated that the species in the subfamily *Erynioideae* showed a strong tendency to infect species in the order Hemiptera. However, our results indicate that equal numbers of species in the *Erynioideae* infect Diptera as infect Hemiptera. The disagreement between the results of these two studies is certainly due to the differential data that were used. We included many more species and only used published host records, while Möckel et al. [7] used records from culture collections as well as the published literature and included many fewer species. 

We investigated whether suborders within Diptera and Hemiptera would differ in the numbers of fungal species infecting them. Within Diptera, we hypothesized that more species might infect nematocerans than brachycerans because the former are more frequently associated with aquatic habitats, and these fungi often require humid conditions for optimal transmission [8]; however, this hypothesis was not supported. Within Hemiptera, we hypothesized that more species might infect Sternorrhyncha because species in this group can be more sessile and aggregated compared to Auchenorrhyncha and Heteroptera, both of these characteristics could improve chances for infection. Indeed, we found that the number of fungal species infecting Sternorrhyncha was the greatest. 

Our major goal was to report the breadth of entomophthoromycotinan host specificity under natural, and not experimental, conditions. Studies focused specifically on the breadth of naturally occurring host ranges for entomophthoromycotinan species are not abundant. Few intensive studies focusing on levels of naturally occurring host specificity have reported both commonly and rarely infected arthropods. For example, in North America, intensive field studies demonstrated that, while *Entomophaga maimaiga* predominantly infects the erebid *Lymantria dispar*, this species is also known to infect a few other species of erebids, a lasiocampid, and a geometrid, although these latter species were infected at very low levels [45,59]. Such examples based on low levels of infection have been included in our study for consistency in reporting. In the majority of the source literature, the relative abundance of fungal pathogens infecting different hosts is not reported. 

Arthropod pathogens in the *Entomophthoromycotina* generally cause acute infections resulting in host death, and the predominant way that these pathogens are found is by locating hosts from which the fungus is growing; usually hosts die before spores are produced, and after spore production, most fungal structures soon degrade. Data on host ranges of pathogens in the *Entomophthoromycotina* are therefore based on both the ability of these pathogens to overcome a host and use it for reproduction and the ability of scientists to find specimens before the degradation of these ephemeral fungi. Data on host specificity of *Entomophthoromycotina* are most certainly deficient, based largely on sampling bias [7]. Arthropod host species with the most extensive information are often those of economic importance, as these are sampled more frequently and intensively than biodiverse native species. 

### Diversity of Arthropod-Pathogenic Species in the Entomophthoromycotina

The number of arthropod-pathogenic species in the family *Entomophthoraceae* (178; 81.3% of the total of 219, not including *Tarichium*) far exceeds the numbers in other entomophthoromycotinan families (Table 1). The arthropod-pathogenic species in the remaining five families in the *Entomophthoromycotina* together include 18.7% of the total species of arthropod pathogens (not including *Tarichium*). The 27 species in the form-genus *Tarichium* remain unassigned to the correct genera and families and therefore have not been included in this summarization. 

Gryganskyi et al. [47] hypothesized that arthropod-pathogenic lifestyles in the *Entomophthoromycotina* evolved independently numerous times from the more phylogenetically basal groups, which were originally saprotrophic. Some of the more basal groups in the *Entomophthoromycotina* include groups with no arthropod pathogens (e.g., *Basidiobolales*, *Ancylistes*, *Macrobiotophthora*, *Capillidiaceae*) or include low percentages of arthropod-pathogenic species (e.g., *Conidiobolaceae* and *Neoconidiobolaceae*). Many species within these latter families have been isolated from soils and are assumed to be saprophytic, and relatively few have adapted the ability to sometimes infect arthropods. For example, among the twenty species of *Conidiobolus* (Nie et al. pers. comm.), only six are arthropod pathogens, and among the ten species of *Neoconidiobolus*, only two are arthropod pathogens. *Conidiobolus coronatus*, in particular, has been considered a saprophyte that at times parasitizes diverse groups of arthropods (i.e., in addition to insects, it infects other arthropods associated with soil: collembolans, a tick, and a myriapod) [12,60]. *Conidiobolus coronatus* and *Neoconidiobolus lamprauges* are also unusual because they can switch to vertebrates, infecting mucous membranes (e.g., [61,62]). These two families (*Conidiobolaceae* and *Neoconidiobolaceae*) begin the transition to arthropod pathogenicity occurring in the more advanced families in the *Entomophthoromycotina*, i.e., *Batkoaceae* and then *Entomophthoraceae*, in which all species are pathogens. Species in the *Batkoaceae* appear to be a transition group between the conidiobolus-like families (*Conidiobolaceae*, *Capillidiaceae*, *Neoconidiobolaceae*) and the *Entomophthoraceae*. All species in the *Batkoaceae* are arthropod pathogens, and this adaptation seems to have been a successful adaptation as all *Entomophthoraceae* are pathogens. However, five species of the eleven in the small family *Batkoaceae* (45.5%) have hosts in multiple orders (Table 1), while only 11 of the 178 species in *Entomophthoraceae* (6.2%) have this broader host range. Unfortunately, most species in the *Batkoaceae* are only known from the original descriptions and host breadth has not been explored extensively. Thus, as species in the *Entomophthoromycotina* evolved, they transitioned from a few being arthropod pathogens to all being arthropod pathogens, with a general trend from broader host ranges toward host specialization, although *Z. radicans* in the *Erynioideae* appears to be a reversal. 

The phylogenetic position of the family *Neozygitaceae* within the *Entomophthoromycotina* is not well understood, although at present it has been placed within a class outside of *Entomophthoromycetes* [44,63]. Regardless of where this family is eventually placed phylogenetically, host use differs to some extent between these classes. Of the twenty-one species of arthropod pathogens in the *Neozygitales*, seven infect non-insect arthropods (33.3%), whereas in the *Entomophthorales*, of the 198 species, only 3 infect non-insect arthropods (1.5%) (Figure 5; Table S1). 

The form-genus *Tarichium* will be resolved once either conidial stages are found for these species (which is less likely) or samples with resting spores are found and molecular methods are employed. It has been suggested that species in this group belong either to the *Entomophthoraceae* or the *Neozygitaceae* [63]. In confirmation, two species previously in *Tarichium* were moved to the genus *Zoophthora* in the *Entomophthoraceae*, based on molecular studies [47]. Additionally, confirming this hypothesis, of the 27 species presently in the form-genus *Tarichium*, 17 are known to infect mites, as is characteristic of some species in the *Neozygitaceae* and uncommon for *Entomophthoraceae* (see above), suggesting that some species of *Tarichium* could belong in the *Neozygitaceae* [64]. Regardless, as with other *Entomophthoromycotina*, many of the species of *Tarichium* are each known only from limited sites and specimens, suggesting that, for a better accuracy in host range data, further sampling is necessary. 

## 5. Conclusions

This meta-analysis demonstrates that the majority of arthropod-pathogenic species in the *Entomophthoromycotina* are specialists, most commonly only infecting species within one host family. Generalist pathogens are few and mostly present in the basal families in this fungal subphylum. Most arthropod pathogens in the *Entomophthoromycotina* infect insects, although a few are pathogens of arachnids, myriapods, and collembolans. Most fungal families in this subphylum do not specialize on a particular arthropod order. 

Further in-depth studies detailing host ranges in this subphylum, especially involving the basal fungal families, are needed as very often for many species the host ranges are based only on the hosts from which pathogens are described. Further information about host ranges will improve our understanding of the predominant evolution of specialism within this subphylum. Insights can be used to explore hypotheses regarding the evolution of host shifts, including ecological fitting, adaptive plasticity, and non-adaptive plasticity [65]. Do mechanisms for host shifting differ for specialist versus generalist pathogens? We hypothesize that non-adaptive plasticity could be more characteristic of generalist pathogens as these are able to shift to infect novel or infrequent hosts. Information from such studies could help to predict results from encounters between emerging pathogens and novel hosts, as such encounters occur increasingly with globalization. 

## Figures and Tables

**Table 1:** Percent arthropod-pathogenic species in fungal families and subfamilies within the *Entomophthoromycotina* by host breadth category. The form-genus *Tarichium* is included in this summary although it has been hypothesized as being polyphyletic.

		% Species			
	Total Arthropod Pathogenic Species	1 Host Family	>1 Host Family in the Same Order	2 Host Orders	>2 Host Orders
Class *Neozygitomycetes*					
Order *Neozygitales*					
Family *Neozygitaceae*	21	90.4%	4.8%	4.8%	0.0%
Class *Entomophthoromycetes*					
Order *Entomophthorales* ^1^					
Family *Conidiobolaceae* ^2^	6	83.3%	0.0%	0.0%	16.7%
Family *Neoconidiobolaceae* ^2^	2	0.0%	0.0%	0.0%	100.0%
Family *Batkoaceae*	11	54.5%	0.0%	27.3%	18.2%
Family *Meristacraceae*	1	100%	0.0%	0.0%	0.0%
Family *Entomophthoraceae*					
Subfamily *Erynioideae*	110	74.8%	17.1%	7.2%	0.9%
Subfamily *Entomophthoroideae*	68	77.6%	19.4%	3.0%	0.0%
Form Genus					
*Tarichium*	27	77.8%	18.5%	3.7%	0.0%

^1^ Families within the *Entomophthorales* are based on the recent division of *Ancylistaceae* reported in Gryganskyi et al. [47]. Higher level taxonomy as presented in Möckel et al. [7]. ^2^ Previously in the family *Ancylistaceae*.

## Data Availability

Data used in this study are available in the Supplementary Materials.

## Supplementary Materials

The following supporting information can be downloaded at: https://www.mdpi.com/article/10.3390/microorganisms11071658/s1, Table S1: Arthropod hosts associated with entomophthoromycotinan species; Table S2: A. Entomophthoromycotinan species excluded based on uncertain fungal taxonomy. B. Entomophthoromycotinan species excluded based on insufficient host identification, Supplementary Material, SM1: References associated with fungal/host data are in Table S1.
