# Supplementary material for: Diversity and Breadth of Host Specificity among Arthropod Pathogens in the Entomophthoromycotina
Source: Microorganisms. 2023 Jun 26;11(7):1658. doi: 10.3390/microorganisms11071658 (PMC10386553; doi:10.3390/microorganisms11071658)
Supplement: Supplementary file 1 [file microorganisms-11-01658-s001.zip › Supplementary Table S2.Apr26.pdf]

Supplementary Table S2A: Species excluded based on uncertain fungal taxonomy.

Species	Reason for exclusion
Entomophthora arrenoctona	Possible synonym of *Eryniopsis caroliniana*
Entomophthora pelliculosa	Possible synonym of *Entomophthora muscae*
Massospora platypediae	Synonym of *Massospora levispora*
Entomophthora egressa	Possible synonym
Entomophthora lauxaniae	Possible synonym
Entomophthora pyralidarum	Possible synonym
Entomophthora oehrensiana	Species name in question
Entomophthora rimosa	Species name in question
Entomophthora schroeteri	Species name in question
Zoophthora exitialis	Species name in question
Entomophthora jassi	Uncertain species status
Entomophthora plusiae	Uncertain species status

Supplementary Table S2B: Species excluded based on insufficient host identification.

Species	Reason for exclusion
Tarichium tatricum	Found in unspecified arachnid
Entomophthora staritzii	Found in unspecified insect
Entomophthora coleopterorum	Found in unspecified Coleoptera
Entomophthora reticulata	Found in unspecified Diptera
Entomophthora richteri	Found in unspecified Diptera
Erynia fluvialis	Found in unspecified Diptera
Erynia gracilis	Found in unspecified Diptera
Furia fumimontana	Found in unspecified Diptera
Neoconidiobolus stromoideus	Found in unspecified Diptera
Pandora muscivora	Found in unspecified Diptera
Tarichium subpunctulatum	Found in unspecified Diptera
Zoophthora obtusa	Found in unspecified Diptera
Entomophthora colorata	Found in unspecified Orthoptera
