# Supplementary material for: Diversity and Breadth of Host Specificity among Arthropod Pathogens in the Entomophthoromycotina
Source: Microorganisms. 2023 Jun 26;11(7):1658. doi: 10.3390/microorganisms11071658 (PMC10386553; doi:10.3390/microorganisms11071658)
Supplement: Supplementary file 1 [file microorganisms-11-01658-s001.zip › SupplementaryMaterials1.AEH.June25.pdf]

References for Supplementary Table S1

Acosta, R.I.T., Humber, R.A., Sánchez-Peña, S.R., 2016. *Zoophthora radicans* (Entomophthorales), a fungal pathogen of *Bagrada hilaris* and *Bactericera cockerelli* (Hemiptera: Pentatomidae and Triozidae): prevalence, pathogenicity, and interplay of environmental influence, morphology, and sequence data on fungal identification. J. Invertebr. Pathol. 139, 82-91.

Anderson, J.F., Ringo, S.L., 1969. *Entomophthora aquatica* sp. n. infecting larvae and pupae of floodwater mosquitoes. J. Invertebr. Pathol. 13(3), 386-393.

Andreasen M., Möller E., Fjelde M., 2021. *Neozygites sminthuri* (Entomophthoromycota, Neozygitales) a fungal pathogen on springtails new to Norway. Agarica 42, 133-138.

Bałazy S., Wiśniewski, J., 1982. Two new species of entomopathogenic fungi on the myrmecophilic mite *Trachhyurpoda caccinea* (Michael, 1891) (Acari: Uropodinae). Bull. Acad. Pol. Sci. 30, 81-84.

Bałazy S., Wiśniewski, J., 1984. Records on some lower fungi occurring in mites (Acarina) from Poland. Acta Mycol. 20(2), 159-172. https://doi.org/10.5586/am.1984.013

Bałazy, S., 1982. New species of Entomophthoraceae from the Wielkopolski National Park. Bull. Acad. Pol. Sci. Ser. Sci. Biol. 29, 221-226.

Bałazy, S., 1993. Flora of Poland. Fungi (Mycota) vol. XXIV. Entomophthorales. Polska Akademia Nauk. Instytut Botaniki im. W. Szafera. Kraków.

Bałazy, S., 2003. On some little known epizootics in noxious and beneficial arthropod populations cause by entomophthoralean fungi. IOBC WPRS Bull. 26(1), 63-68.

Bałazy, S., Wiśniewski, J., Kaczmarek, S., 1987. Some noteworthy fungi occurring on mites. Bull. Acad. Pol. Sci., Ser. Sci, Biol. 35, 199-224.

Barta, M., Cagáň, L., 2003. *Pandora uroleuconii* sp.nov. (Zygomycetes: Entomopthoraceae), a new pathogen of aphids. Mycotaxon 88, 79-86.

Ben-Ze'ev I., Kenneth R.G., 1979. *Zoophthora erinacea* sp. n. [new species] (Zygomycetes: Entomophthoraceae), a fungal parasite of aphids [*Aphis umbrella, Aphis fabae, Myzus persicae*] in an alfalfa field near Rehovot, in the Coastal Plain of Israel. Mycotaxon 10(1), 219-232.

Ben-Ze'ev I., Kenneth R.G., 1980. *Zoophthora phytonomi* and *Conidiobolus osmodes* [Zygomycetes: Entomophthoraceae], two pathogens of *Hypera* species [Col.: Curculionidae] coincidental in time and place. Entomophaga 25(2), 171-186.

Ben-Ze'ev I., Kenneth R.G., 1981a. *Zoophthora orientalis* sp. nov., a fungal pathogen of *Aphis citricola* (Homoptera: Aphidiade), and two new combinations of other species of Entomophthoraceae. Phytoparasitica 9(1), 33-42.

Ben-Ze'ev I., Kenneth R.G., 1981b. Zoophthora radicans and *Zoophthora petchi* sp. nov. [Zygomycetes: Entomophthorales], two species of the "sphaerosperma group" attacking leaf-hoppers and frog-hoppers [Hom.]. Entomophaga 26(2), 131-142.

Ben-Ze'ev, I.S., Kenneth, R.G., Uziel, A., 1987. A reclassification of *Entomophthora turbinata* in Thaxterosporium gen. nov., Neozygitaceae fam. nov. (Zygomycetes: Entomophthorales). Mycotaxon 28, 313-326.

Ben-Ze'ev, I., Keller, S., Ewen, A.B., 1985. *Entomophthora erupta* and *Entomophthora helvetica* sp. nov. (Zygomycetes, Entomophthorales) two pathogens of Miridae (Heteroptera) distinguished by pathological and nuclear features. Can. J. Bot. 63, 1459-1475.

Ben-Ze'ev, I.S., Zelig, Y., 1984. *Entomophthora israelensis* sp. nov. (Zygomycetes: Entomophthorales), a fungal pathogen of gall midges (Diptera: Cecidomyiidae). Mycotaxon 21, 463-474.

Ben-Ze'ev, I.S., Zelig, Y., Bitton, S., Kenneth, R.G., 1988. The Entomophthorales of Israel and their arthropod hosts: Additions 1980-1988. Phytoparasitica 16(3), 247-257.

Cuebas-Incle, E.L., 1992. Infection of adult mosquitoes by the entomopathogenic fungus *Erynia conica* (Entomophthorales: Entomophthoraceae). J. Am. Mosq. Contr. Assoc. 8(4), 367-371.

Delalibera Jr., I., Hajek, A.E., Humber, R.A., 2004. *Neozygites tanajoae*, sp. nov., a pathogen of the cassava green mite. Mycologia 96(5), 1002-1009. https://doi.org/10.1080/15572536.2005.11832900

Descals, E., Webster, J., 1984. Branched aquatic conidia in *Erynia* and *Entomophthora* sensu lato. Trans. Brit. Mycol. Soc. 83, 669-682.

Descals, E., Webster, J., Ladle, M., Bas, J.A.B, 1981. Variations in asexual reproduction in species of *Entomophthora* on aquatic insects. Trans. Brit. Mycol. Soc. 77, 85-102.

Dromph, K.M., Eilenberg, J., Esbjerg, P., 2001. Natural occurrence of entomophthoralean fungi pathogenic to Collembolans. J. Invertebr. Pathol. 78, 226-231.

Eilenberg, J., Michelsen, V., Humber, R.A., 2020. *Strongwellsea tigrinae* and *Strongwellsea acerosa* (Entomophthorales: Entomophthoraceae), two new species infecting dipteran hosts from

the genus *Coenosia* (Muscidae). J. Invertebr. Pathol. 175, 107444. https://doi.org/10.1016/j.jip.2020.107444

Eilenberg, J., Michelson, V., Jensen, A.B., Humber, R.A., 2021. *Strongwellsea crypta* (Entomophthorales: Entomophthoraceae), a new species infecting *Botanophila fugax* (Diptera: Anthomyiidae). J. Invertebr. Pathol. 186, 107673. https://doi.org/10.1016/j.jip.2021.107673

Eilenberg, J., Michelsen, V., Jensen, A.B., Humber, R.A., 2022. *Strongwellsea selandia* and *Strongwellsea gefion* (Entomophthorales: Entomophthoraceae), two new species infecting adult flies from genus *Helina* (Diptera: Muscidae). J. Invertebr. Pathol. 193, 107797. https://doi.org/10.1016/j.jip.2022.107797

Fan, M.Z., Li, Z. Z., 1994. Two new pathogens of dipteran insects. Mycotaxon 50, 309-314.

Feng, M., Chen, C., Chen, B., 2004. Wide dispersal of aphid-pathogenic Entomophthorales among aphids relies upon migratory alates. Environ. Microbiol. 6(5), 510-516.

Filotas, M.J.F., Hajek, A.E., Humber, R.A., 2003. Prevalence and biology of *Furia gastropachae* (Zygomycetes: Entomophthorales) in populations of the forest tent caterpillar (Lepidoptera: Lasiocampidae). Can. Entomol. 135, 359-378.

Folgarait, P.J., Goffré, D., 2021, *Conidiobolus lunulus*, a newly discovered entomophthoralean species, pathogenic and specific to leaf-cutter ants. J. Invertebr. Pathol. 186, 107685. https://doi.org/10.1016/j.jip.2021.107685

Gindin, G., Ben-Ze'ev, I.S., 1994. Natural occurrence of and inoculation experiments with *Conidiobolus coronatus* and *Conidiobolus* sp. in glasshouse populations of *Bemisia tabaci*. Phytoparasitica 22(3), 197-208.

Gokce, A., Er, M.K., 2003. First description of the disease of *Conidiobolus osmodes* on *Tipula paludosa* larvae with the report of a natural epizootic. J. Invertebr. Pathol. 84, 83-89.

Gouli, V.V., Marcelino, J.A., Gouli, S.Y., 2020. Microbial Pesticides: Biological Resources, Production and Application. Academic Press.

Gres, J.A., Koval, E.Z., 1982. *Entomophthora terrestris* sp. nov. affecting the sugar beet aphid. Microbiol. J. 44, 64-69. (in Russian)

Gryganskyi, A., Golan, J., Hajek, A.E., 2022. Season-long infection of diverse hosts by the entomopathogenic fungus *Batkoa major.* PLoS ONE 17, e0261912.

Hajek, A.E., Butler, L., Walsh, S.R.A., Silver, J.C., Hain, F.P., Hastings, F.L., ODell, T.M., Smitley, D.R., 1996. Host range of the gypsy moth (Lepidoptera: Lymantriidae) pathogen *Entomophaga maimaiga* (Zygomycetes: Entomophthorales) in the field versus laboratory. Environ. Entomol. 25, 709-721.

Hajek, A.E., Butler, L., Liebherr, J.K., Wheeler, M.M., 2000. Risk of infection by the fungal pathogen *Entomophaga maimaiga* among Lepidoptera on the forest floor. Environ. Entomol. 29, 645-650.

Hajek A.E., Gryganskyi A., Bittner T., Liebherr J.K., Liebherr J.H., Jensen A.B., Moulton J.K., Humber R.A., 2016. Phylogenetic placement of two species known only from resting spores: *Zoophthora independentia* sp. nov. and *Z. porteri* comb nov. (Entomophthorales: Entomophthoraceae). J. Invertebr. Pathol. 140, 68-74. https://doi.org/10.1016/j.jip.2016.09.002

Hajek, A.E., Jensen, A.B., Thomsen, L., Hodge, K.T., Eilenberg, J., 2003. PCR-RFLP is used to investigate relations among species in the entomopathogenic genera *Eryniopsis* and *Entomophaga*. Mycologia 95(2), 262-268. https://doi.org/10.1016/j.jip.2016.09.002

Hannam, J.J., Steinkraus, D.C., 2010. The natural occurrence of *Pandora heteropterae* (Zygomycetes: Entomophthorales) infecting *Lygus lineolaris* (Hemiptera: Miridae). J. Invertebr. Pathol. 103, 96-102.

Hodge, K.T., Hajek, A.E., 2017. The first entomophthoralean killing millipedes, *Arthrophaga myriapodina* n. gen. n. sp., causes climbing before host death. J. Invertebr. Pathol. 149, 135-140. https://doi.org/10.1016/j.jip.2017.08.011

Huang, B., Humber, R., Hodge, K.T., 2007. A new species of *Conidiobolus* from Great Smoky Mountains National Park. Mycotaxon 100, 227-233.

Huang, Y.J., Li, Z.Z., 1993. *Furia fujiana*, a new pathogen of pale-lined tiger moth, *Spilarctia obliqua*. Mycosystema 12(1), 1-4.

Humber, R.A., 1981. An alternative view of certain taxonomic criteria used in the Entomophthorales (Zygomycetes). Mycotaxon 13(1), 191-240.

Humber, R.A., 1984a. *Eryniopsis*, a new genus of the Entomophthoraceae (Entomophthorales). Mycotaxon 21, 257-264.

Humber, R.A., 1984b. The identity of *Entomophaga* species (Entomophthorales: Entomophthoraceae) attacking Lepidoptera. Mycotaxon 21, 265-272.

Humber, R.A., Ben-Ze'ev, I., 1981. *Erynia* (Zygomycetes: Entomophthorales): emendation, synonymy, and transfers. Mycotaxon 13(3), 506-516.

Jensen, A.B., Thomsen, L., Eilenberg, J., 2006. Value of host range, morphological, and genetic characteristics within the *Entomophthora* muscae species complex. Mycol. Res. 110, 941-950.

Keller, S., 1978. *Entomophthora gigantea* sp. n. and *E. caroliniana* (Thaxter) comb. nov., two pathogens of *Tipula paludosa* Meig. Sydowia 31, 87-93.

Keller, S., 1980. Two new species of the genus *Zoophthora* Batko (Zygomycetes, Entomophthoraceae): *Z. lanceolata* and *Z. crassitunicata*. Sydowia 33, 167-170.

Keller, S., 1987. Arthropod-pathogenic Entomophthorales of Switzerland I. *Conidiobolus*, *Entomophaga* and *Entomophthora*. Sydowia 40, 122-167.

Keller, S., 1991. Arthropod-pathogenic Entomophthorales of Switzerland. II. *Erynia, Neozygites*, *Zoophthora*, and *Tarichium.* Sydowia 43, 39-122.

Keller, S., 1994. Validation of the description of some species of Entomophthorales (Zygomycetes). Sydowia 46(1), 41-43.

Keller, S., 1997. The genus *Neozygites* (Zygomycetes, Entomophthorales) with special reference to species found in tropical regions. Sydowia 46(2), 118-146.

Keller, S., 2002. The genus *Entomophthora* (Zygomycetes, Entomophthorales) with a description of five new species. Sydowia 56(2), 241-242

Keller, S., 2006. Species of Entomophthorales attacking aphids with description of two new species. Sydowia 58(1), 38-74.

Keller, S., 2007. Arthropod-pathogenic Entomophthorales from Switzerland. III. First additions. Sydowia 59(1), 75-113.

Keller, S., 2011. *Pandora psocopterae*: a new species of insect-pathogenic Entomophthoraceae (Fungi, Entomophthoromycetes) from France. Mitt. Schweiz. Ent. Ges. 84, 181-183.

Keller, S., 2012. Arthropod-pathogenic Entomophthorales from Switzerland. IV. Second addition. Mitt. Schweiz. Ent. Ges. 85, 115-130.

Keller, S., 2013. Entomophthorales (Fungi, Entomophthoromycota) attacking Coleoptera with a key for their identification, Mitt. Schweiz. Ent. Ges. 86, 261-279.

Keller, S., 2018. Amended description and new combination for *Entomophthora nebriae* Raunkiaer, (1893), a little known entomopathogenic fungus attacking the ground beetle *Nebria brevicollis* (Fabricius, 1792). Alpine Entomol. 2, 1-5. https://doi.org/10.3897/alpento.2.22136

Keller, S., Steenberg, T., 1997. *Neozygites sminthuri* sp. nov. (Zygomycetes, Entomophthorales), a pathogen of the springtail *Sminthurus viridis* L. (Collembola, Sminthuridae). Sydowia 49, 21-24.

Keller, S., Weiser, J., Wegensteiner, R., 2009. *Tarichium hylobii* sp. nov., a pathogen of *Hylobius abietis*. Sydowia 61(2), 249-254.

Keller, S., Hülsewig, T., Jensen, A.B., 2022. Fungi attacking springtails (Sminthuridae, Collembola) with a description of *Pandora batallata*, sp. nov. (Entomophthoraceae). Sydowia, 75, 37-45.

Kermarrec, A., Mauleon, H., 1975. Quelques aspects de la pathogénie d'*Entomophthora coronata* Cost. Kervork. pour la Fourmi-Manioc de la Guadeloupe: *Acromyrmex octospinosus* (Formicidae, Attini). Ann. Parasitol. Hum. Comp. 50(3), 351-360.

Kramer, J.P., 1981. A mycosis of the blood-sucking snipe fly *Symphoromyia hirta* caused by *Erynia ithacensis* sp. n. (Entomophthoraceae). Mycopathologia 75, 159-164.

Latchininsky, A.V., Temreshev, I.I., Childebaev, M.K., Kolov, S.V., 2016. Host range and recorded distribution of the fungal pathogen *Entomophaga grylli* (Entomophthoromycota: Entomophthorales) in Kazakhstan. J. Orthop. Res. 25(2), 83-89.

Leatherdale, D., 1970. The arthropod hosts of entomogenous fungi in Britain. Entomophaga 15, 419-435.

Li, Z.Z., 1986. *Erynia anhuiensis*, a new pathogen of aphids. Acta Mycol. Sinica 5(1), 1-6. (in Chinese)

Li, Z.Z., 2000. Flora Fungorum Sinicorum, vol. 13, Entomophthorales. Beijing, China: Science Press.

Li, Z.Z., Huang, B., Fan, M.Z., 1997. New species, new records, new combinations and emendation of entomophthoralean fungi pathogenic on dipteran insects. Mycosystema 16(2), 91–96.

Li Z.Z., Fan M.Z., Huang B., 1998. New combinations of entomophthoralean fungi originally in the genus *Erynia*. Mycosystema 7(1), 91-94.

López-L., C.C., Toledo, A.V., Manfrino, R.G., Gutierrez, A.C., 2019. Southernmost records of Entomophthoromycotina. Updated review of entomophthoralean fungal insect pathogens of Argentina. Caldasia 41(2), 349-357.

Macias A.M., Geiser D.M., Stajich J.E., Łukasik P., Veloso C., Bublitz D.C., Berger M.C., Boyce G.R., Hodge K., Kasson M.T., 2020. Evolutionary relationships among *Massospora* spp. (Entomophthorales), obligate pathogens of cicadas. Mycologia 112(6), 1060-1074.

MacLeod, D.M., Müller-Kögler, E., 1973. Entomogenous fungi: *Entomophthora* species with pear-shaped to almost spherical conidia (Entomophthorales: Entomophthoraceae). Mycologia 65, 825-893.

Małagocka, J., Jensen, A.B. and Eilenberg, J., 2017. *Pandora formicae*, a specialist ant pathogenic fungus: new insights into biology and taxonomy. J. Invert. Pathol. 143, 108-114.

Mascarin, G.M., da Silveira Duarte, V., Brandão, M.M., Delalibera Jr., I., 2012. Natural occurrence of *Zoophthora radicans* (Entomophthorales: Entomophthoraceae) on *Thaumastocoris peregrinus* (Heteroptera: Thaumastocoridae), an invasive pest recently found in Brazil. J. Invertebr. Pathol. 110(3), 401-404. doi: 10.1016/j.jip.2012.03.025.

Matanmi, B.A., Libby, J.L., Maxwell, D.P., 1974. Two Phycomycetes infecting root maggot adults in Wisconsin. Environ. Entomol. 3, 1030-1031.

Meija, B.S., Keller, S., 2003. *Entomophthora leyteensis* Villacarlos & Keller sp. nov. (Entomophthorales: Zygomycetes) infecting *Tetraleurodes acaciae* (Quaintance) (Insecta, Hemiptera; Aleyrodidae), a recently introduced whitefly on *Gliricida sepium* (Jaq.) Walp. (Fabaceae) in the Philippines. J. Invertebr. Pathol. 83, 16-22.

Miętkiewski, R., Bałazy, S., Tkaczuk, C., 2000. Mycopathogens of mites in Poland-A review. Biocontr. Sci. Technol. 10(4), 459-65. https://doi.org/10.1080/09583150050115043

Miętkiewski, R., Bałazy, S., 2003. *Neozygites abacaridis* sp. nov. (Entomophthorales), a new pathogen of phytophagous mites (Acari, Eriophyidae). J. Invertebr. Pathol. 83(3), 223-229.

Milner, R.J., 1986. *Neozygites acaridis* (Petch) comb. nov.: An entomophthoran pathogen of the mite, *Macrocheles peregrinus*, in Australia. Trans. Br. Mycol. Soc. 85(4), 641-647.

Montalva, C., Arismendi, N., Barta, M., Rojas, E., 2013. Molecular differentiation of recently described *Neozygites osornensis* (Neozygitales: Neozygitaceae) from two morphologically similar species. J. Invertebr. Pathol. 115, 92-94. https://doi.org/10.1016/j.jip.2013.10.007

Montalva, C., Rocha, L., Fernandes, É., Luz, C., Humber, R., 2016. *Conidiobolus macrosporus* (Entomophthorales), a mosquito pathogen in Central Brazil. J. Invert. Pathol. 139, 102-108. https://doi.org/10.1016/j.jip.2016.08.003

Nadeau, M.P., Dunphy, G.B., Boisvert, J.L., 1994. Entomopathogenic fungi of the order Entomophthorales (Zygomycotina) in adult black fly populations (Diptera: Simuliidae) in Quebec. Can. J. Microbiol. 40(8), 682-686.

Nie, Y., Yu, D.S., Wang, C.F., Liu, X.Y., Huang, B., 2020. A taxonomic revision of the genus *Conidiobolus* (Ancylistaceae, Entomophthorales): four clades including three new genera. MycoKeys 66, 55. 10.3897/mycokeys.66.46575

Niell, M., Santamaria, S., 2001. Additions to the knowledge of entomopathogenic Entomophthorales (Fungi, Zygomycota) from Spain. Nova Hedwig. 73(1-2), 167-184.

Remaudière, G., Hennebert, G.L., 1980. Revision systematique de *Entomophthora aphidis* Hoffm. in Fres. Description de deux nouveaux pathogenes d'aphides. Mycotaxon 11(1), 269-321.

Remaudière, G., Keller, S., 1980. Révision systématique des genres d'Entomophthoraceae à potentialité entomopathogène. Mycotaxon 11(1), 330-331.

Samsináková, A., Kálalová, S., Daniel, M., Dusbábek, F., Honzáková, E., Cerný, V. 1974. Entomogenous fungi associated with the tick *Ixodes ricinus*. Fol. Parasitol. 21, 39-48.

Soper, R.S., Shimazu, M., Humber, R.A., Ramos, M.E., Hajek, A.E., 1988. Isolation and characterization of *Entomophaga maimaiga* sp. nov., a fungal pathogen of gypsy moth, *Lymantria dispar*, from Japan. J. Invertebr. Pathol. 51, 229-241.

Sosa Gomez, D.R., Lopez Lastra, C.C., Humber, R.A., 2010. An overview of arthropod-associated fungi from Argentina and Brazil. Mycopathologia 170, 61-76.

Steinkraus, D.C., Kramer, J.P., 1989. Development of resting spores of *Erynia aquatica* (Zygomycetes: Entomophthoraceae) in *Aedes aegypti* (Diptera: Culicidae). Environ. Entomol. 18(6), 1147-1152. https://doi.org/10.1093/ee/18.6.1147

Steinkraus, D.C., Oliver, J.B., Humber, R.A., Gaylor, M.J., 1998. Mycosis of bandedwinged whitefly (*Trialeurodes abutilonea*) (Homoptera: Aleyrodidae) caused by *Orthomyces aleyrodis* gen. & sp. nov. (Entomophthorales: Entomophthoraceae). J. Invertebr. Pathol. 72(1), 1-8.

Stimmann, M.W., 1968. Effect of temperature on infection of the garden symphylan by *Entomophthora coronata*. J. Econ. Entomol. 61(6), 1558-1560.

Tkaczuk, C., Bałazy, S., Krzyczkowski, T., Wegensteiner, R., 2011. Extended studies on the diversity of arthropod-pathogenic fungi in Austria and Poland. Acta Mycologica 46, 211-222.

Villacarlos, L.T., Keller, S., 1997. *Batkoa amrascae* Keller & Villacarlos, a new species of Entomophthorales (Zygomycetes) infecting the cotton leafhopper, *Amrasca biguttula* (Ishida) (Homoptera: Cicadellidae) in the Philippines. Philipp. Entomol. 11(1), 81-86.

Villacarlos, L., Wilding N., 1994. Four new species of Entomophthorales infecting the leucaena psyllid, *Heteropsylla cubana*, in the Philippines. Mycol. Res. 98(2), 153-64.

Wang, W., Lu, W., Li, Z., 1994. *Furia shandongensis* (Zygomycetes: Entomophthorales), a new pathogen of earwigs. Mycotaxon 50, 301-306.

Waterhouse, G.M., Brady, B.L., 1982. Key to the species of *Entomophthora* sensu lato. Bull. Brit. Mycol. Soc. 16(2), 113-43.

Zha, L., Wen, T., Hyde, K., Kang, J., 2016. An updated checklist of fungal species in Entomophthorales and their host insects from China. Mycosystema 6, 666-683. 10.13346/j.mycosystema.150010

Zhou, X., Montalva, C., Arismendi, N., Hong, F., 2017. *Neozygites linanensis* sp. nov., a fungal pathogen infecting bamboo aphids in southeast China. Mycotaxon 132(2), 305-315. https://doi.org/10.5248/132.305
